# Structures of Biological Minerals in Dental Research

**DOI:** 10.6028/jres.106.054

**Published:** 2001-12-01

**Authors:** Mathai Mathew, Shozo Takagi

**Affiliations:** American Dental Association Health Foundation, Paffenbarger Research Center, National Institute of Standards and Technology, Gaithersburg, MD 20899-0001 USA

**Keywords:** biominerals, calcium phosphates, crystal structure, fluorapatite, glaserite, hydroxyapatite, octacalcium phosphate

## Abstract

Structural features of some calcium phosphates of biological interest are described. Structure of hydroxyapatite (OHAp), considered as the prototype for the inorganic component of bones and teeth is discussed with respect to the kinds and locations of ionic substitutions. Octacalcium phosphate (OCP), is a probable precursor in biological mineralization. OCP has a layer type structure, with one layer quite similar to that of OHAp and the other, a hydrated layer consisting of more widely spaced Ca, and PO_4_ ions and the water molecules. The closeness of fit in the apatitic layers of OCP and OHAp accounts for the epitaxial, interlayered mixtures formed by these compounds and the in situ conversion of OCP to OHAp. Possible roles of OCP in biological mineralization are discussed.

## 1. Introduction

The crystallography program at the American Dental Association Health Foundation, Paffenbarger Research Center, was initiated in the early 1970s. The program was designed to investigate the crystal structures of biological minerals and related compounds associated with or with potential bearing in mineralization processes. Since calcium phosphates comprise the largest group of biominerals in vertebrate animals, most of the work carried out, was centered on or around calcium phosphates or related materials. Crystal structures of a number of pyrophosphates, carbonates, bisphosphonates, and highly hydrated phosphates and arsenates of calcium, magnesium and strontium were also investigated. This review will summarize the structural features of calcium phosphates relevant to biomineralization. The phosphates containing HPO_4_^2−^ and PO_4_^3−^ generally constitute the biologically relevant calcium phosphates. Phosphates with only H_2_PO_4_^−^ ions are not normally found under physiological conditions, but are commercially important as components in fertilizers.

### 2. Discussion—Calcium Phosphates

The known pure calcium phosphates have been classified into three major structural types [1]: (i) the apatite type, Ca_10_(PO_4_)_6_X_2_, which includes the derivatives of hydroxyapatite (X = OH^−^) and fluorapatite (X = F^−^) as well as those related to apatite-type structures such as octacalcium phosphate (OCP), [Octacalcium bis(hydrogenphosphate) tetrakis(phosphate) pentahydrate], Ca_8_(HPO_4_)_2_(PO_4_)_4_·5H_2_O and tetracalcium phosphate (TTCP), Ca_4_(PO_4_)_2_O; (ii) the glaserite type, which can be considered to include all polymorphs of tricalcium phosphates (TCP), Ca_3_(PO_4_)_2_; and (iii) the Ca-PO_4_ sheet-containing compounds, which include dicalcium phosphate dihydrate (DCPD), CaHPO_4_·2H_2_O, dicalcium phosphate anhydrous (DCPA), CaHPO_4_, and monocalcium phosphates, Ca(H_2_PO_4_)_2_·H_2_O and Ca(H_2_PO_4_)_2_. A number of highly hydrated phosphatic compounds have been included as a new type of calcium phosphate [1], struvite-type structures, after the biomineral struvite, Mg(NH_4_)PO_4_·6H_2_O, although these compounds do not represent pure calcium phosphates. Amorphous calcium phosphate (ACP), a possible precursor to bioapatite, may be related to one or more of the structural types discussed above.

### 2.1 Apatite Type Structures

Apatites are a structural type for compounds of the general formula M_10_(XO_4_)_6_Y_2_ rather than specific compounds. In general, they are known to be capable of accommodating a wide variety of modifications and combinations of substitutions of ions and groups within the apatitic lattice. However, the term “apatite” has been extensively and synonymously used to represent the calcium phosphates, Ca_10_(PO_4_)_6_X_2_, where X = F^−^, OH^−^, or Cl^−^·and this concept will be followed in this review. Apatites are thermodynamically the most stable phases among the calcium phosphates and, therefore, can be considered as the probable end product in many reactions.

#### 2.1.1 Hydroxyapatite

Hydroxyapatite (OHAp), Ca_10_(PO_4_)_6_(OH)_2_, is used as a model for inorganic component of bones and teeth. However, apatites as they occur in biological tissues, mineral formations and laboratory products can incorporate a wide variety of impurities and are seldom found in pure stoichiometric form.

**Fig. 1:**
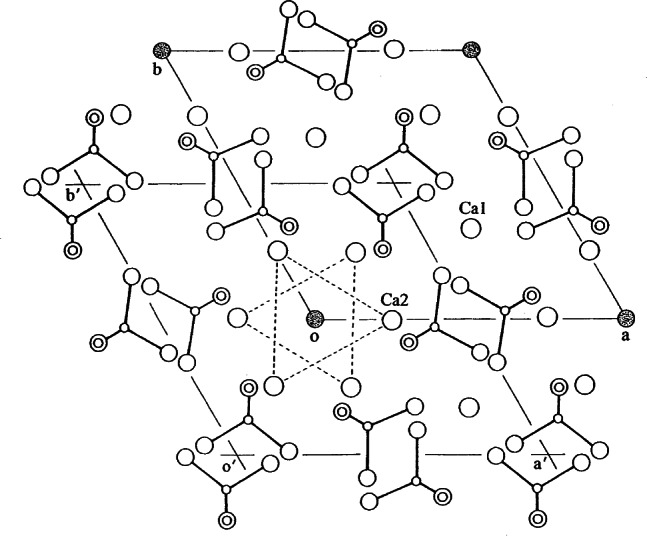
Crystal structure of FAp or hexagonal OHAp projected down the *c*-axis. The corners of the unit cell (marked by shaded circles) are occupied by F^−^ in FAp and by OH^−^ in OHAp. An alternate choice of unit cell is identified as *a*′ and *b*′.

The most common form is hexagonal and the crystal structure has been described in the space group *P*6_3_/*m* (No. 176) with lattice parameters *a* = *b* = 9.432 Å and *c* = 6.881 Å, *Z* = 1 [2]. The structure is depicted in Fig. 1. The 10 Ca^2+^ ions occupy two crystallographically different symmetry sites, 4*f* and 6*h*. Four Ca^2+^ ions (4*f*) are located in columns along the three-fold axes at 1/3, 2/3, 0 and 2/3, 1/3, 0 separated by approximately one-half of the *c*-axis. These are commonly referred to as Ca1 (or column Ca). Ca1 is coordinated to nine O atoms, with six shorter bonds that define an approximate trigonal prism and three longer bonds capping the prism faces. The Ca-O_9_ polyhedra share the trigonal faces to form chains parallel to the *c*-axis. The remaining six Ca^2+^ ions (6*h* sites, referred to as Ca2 or triangular Ca) form two triangular sets at *z* = 1/4 and 3/4 on the mirror planes. The Ca2 ions are seven-coordinated, with six O atoms and one OH^−^ ion. The six PO_4_^3−^ ions occupy 6*h* positions similar to the Ca2 ions, in expanded triangular positions. Adjacent Ca1 and Ca2 polyhedra are linked through oxygen atoms of the PO_4_^3−^ tetrahedra. Because of the crystallographic mirror symmetry imposed by the space group, each OH^−^ ion has to be considered at statistically disordered positions (4*e*) both above and below the mirror planes at *z* = 1/4 and 3/4. It has been shown by neutron diffraction studies [2] that the oxygen atoms in hydroxide ions are 0.34 Å away from the mirror plane with the OH^−^ direction pointing away from the mirror planes. An averaged structure could imply that in approximately half the unit cells the OH−^·^ ions are pointed upward from the mirror plane and in the remaining unit cells they are pointed downward. However, this statistical disordering need not be completely random. At least some short range ordering is to be invoked such as OH-OH-OH…HO-HO. The reversal of the OH^−·^ direction can be achieved by replacement of an OH^−·^ by F^−·^ or Cl^−·^ etc. or by a vacancy. Thus, the hexagonal OHAp is probably never strictly stoichiometric.

Stoichiometric OHAp has been described as monoclinic, space group *P*2_1_/*b* having cell parameters *a* = 9.4214(8) Å, *b* = 2*a, c* = 6.8814(7) Å, *γ*= 120°, with twice as many formula units per unit cell as in the hexagonal unit [3]. The structure is closely related to that of the hexagonal form, but with no restrictions imposed by the mirror symmetry. The Ca^2+^ and PO_4_^3−^ ions occupy similar positions as in the hexagonal form. However, the OH^−·^ ions are located in two different columns. Within each column all the OH^−·^ ions have the same direction of displacements from *z* = 1/4 as in the hexagonal form. All the OH^−·^ ions in one column point upward, while those in the other column point downward. Thus, there is no disordering of the OH^−·^ ions in the monoclinic form. The monoclinic form is formed only under favorable thermal conditions.

#### 2.1.2 Fluorapatite

Fluorapatite (FAp), Ca_10_(PO_4_)_6_F_2_, is the most stable among the apatites. FAp is hexagonal with the space group *P*6_3_/*m* and lattice parameters, *a* = *b* = 9.367(1) Å and *c* = 6.884(1) Å, *Z* = 1 [4]. The positions of the two sets of Ca^2+^ ions and the PO_4_^3−^ ions are nearly identical to those of OHAp. However, the F^−·^ ions occupy the center of the Ca2 triangles (6*h* positions), on the mirror planes at *z* = 1/4 and 3/4.

#### 2.1.3 Chlorapatite

Chlorapatite (ClAp), Ca_10_(PO_4_)_6_(Cl)_2_, has been described in the hexagonal space group *P*6_3_/*m*, with cell parameters, *a* = *b* = 9.598(2) Å, *c* = 6.776(4) Å, *Z* = 1 [5]. Like OH^−·^ in OHAp, the Cl^−·^ is also disordered, displaced from the midpoint of the Ca2 triangles, and in positions 1.2 Å above and below the mirror planes. The Cl^−·^ is so far removed from the mirror plane towards the midway point between the two Ca2 triangles, that an additional weak bond develops between the Ca2 and a second Cl^−·^ ion.

Stoichiometric ClAp has also been found to crystallize in the monoclinic space group with space group *P*2_1_/*b* having cell parameters *a* = 9.628(5) Å, *b* = 2*a, c* = 6.764(5) Å, *γ*= 120°, *Z* = 2 [6]. The structure is very similar to the hexagonal one, but the Cl^−·^ ions are ordered in two columns on pseudohexagonal axes as in the case of the monoclinic OHAp.

#### 2.1.4 Substituted Apatites

OHAp can incorporate a wide variety of substitutions for Ca^2+^, PO_4_^3−^, and/or OH^−·^ ions. Substitution of other elements for Ca^2+^ and PO_4_^3−^ is relatively minor in most natural mineral samples. Natural minerals of the composition, Ca_10_(PO_4_)_6_(F,OH,Cl)_2_ exhibit large variations in F^−^, OH^−^, and Cl^−·^ contents. Pure end-members are uncommon in nature, but binary and ternary compositions are widely reported.

Biological apatites are rarely stoichiometric, are usually calcium-deficient, and contain a wide variety of relatively small amounts of other substituent atoms or groups. A large number of proposals have been made to account for the nonstoichiometry of bioapatites. The major cause of nonstoichiometry is the incorporation of impurities, usually substitutionally for Ca, but also interstitially. Both HPO_4_^2−^, and structurally incorporated water occur in some synthetic and biological apatites, but their structural locations are not known.

##### 2.1.4.1 X^−^ Ion Substitution

The X^−^ ion positions in apatites or the “X ion channels”, as they are often referred to, appear to be the sites of a great deal of interesting activity in apatites. The X^−^ ion positions in apatites are substituted by a variety of ions, frequently by OH^−^, F^−^, and Cl^−^, but also by CO_3_^2−^ and O^2−^, or by vacancies or any combination of these. In pure form each X^−^ ion takes up its own particular location, as noted above. However, when two or more of these ions are present at the same time, they interact with each other to produce effects not predicted from the knowledge of the structures of the end-member alone [5,7,8]. The positional *z*-parameters of the X^−·^ ions are shifted from their normal positions in the pure form, but the effects are more pronounced when the larger Cl^−·^ ions are involved. A monoclinic form of a natural ternary apatite, Ca_5_(PO_4_)_3_(F_0.29_, Cl_0.47_, OH_0.24_), space group *P*2_1_/*b*, has been reported [8]. There are two anion columns in the unit cell and both columns contain all three anions. The reduction in symmetry from hexagonal to monoclinic results from ordering of the column anions in each column in one of the two symmetry-equivalent anion sites present in the hexagonal ternary apatite.

##### 2.1.4.2 Carbonate Apatites

Carbonate apatites are of special interest in biological systems as the inorganic component of bone and teeth. There are two generally accepted locations for the CO_3_^2−^ ion in the apatite lattice: one on the hexad axis at the OH^−·^ ion site (type-A) and the other for PO_4_^3−^ (type-B). Type-B substitution would require involvement of additional ions for charge balance. Biological apatites are principally type-B carbonates, but with small amount of type-A [9]. However, neither of these cases has been confirmed by complete structure analysis and the structure of carbonate-apatite remains controversial.

##### 2.1.4.3 Cation Substitution

The incorporation of foreign cations in the apatite lattice is expected to change the bulk properties of the apatite. The structures of a number of synthetic substituted apatites were investigated to evaluate the structural changes associated with the substitution. Lead is known as a “bone seeker” in that it accumulates in bone and tooth mineral. In a lead apatite study, a short Pb-O distance observed indicating a covalent bond may account for this lead incorporation [10]. The structure of calcium-lanthanam apatite shows that cation ordering in apatites are strongly dependent on the properties of the constituent ions [11]. However, in a series of Ba-rare earth-Na apatites [12] the results indicate the substitutions to be unexpectedly complex to derive any general prediction.

### 2.2 Octacalcium Phosphate

**Fig. 2:**
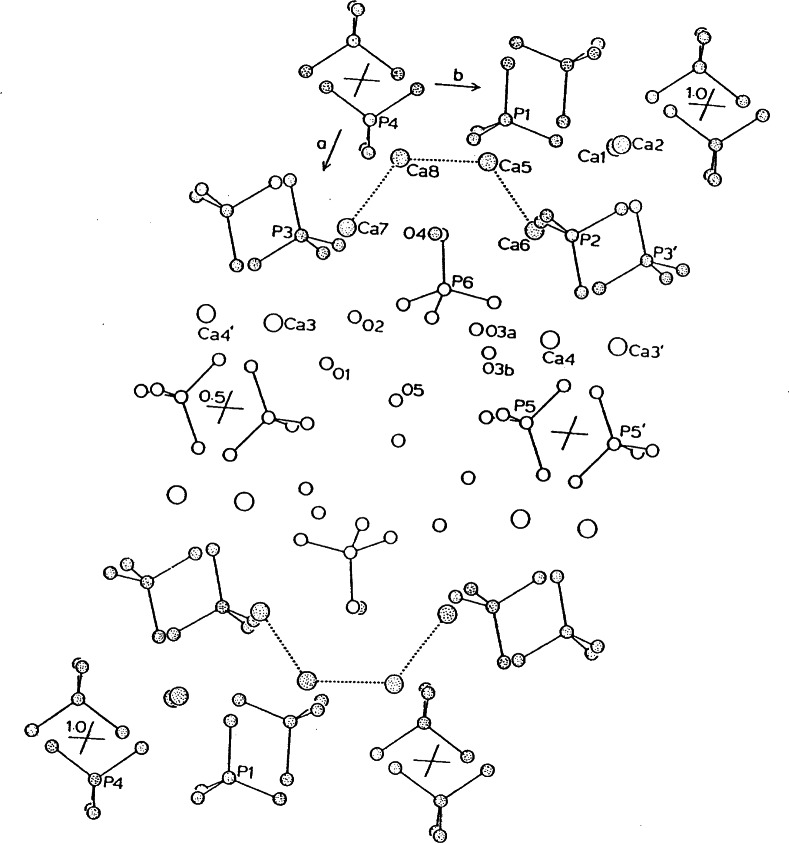
Crystal structure of octacalcium phosphate projected down the *c*-axis. The region with shaded atoms is very similar to that of OHAp and has been referred to as the “apatitic” layer. Hydrogen atoms are omitted for clarity.

The crystal structure of octacalcium phosphate (OCP), Ca_8_(HPO_4_)_2_(PO_4_)_4_·5H_2_O, was initially determined in 1962 [13] and refined later [14]. The crystals are triclinic, space group $P\bar{1}$, with cell parameters *a* = 19.692(4) Å, *b* = 9.523(2) Å, *c* = 6.835(2) Å, *α* = 90.15(2)°, β= 92.54(2)°, = 108.65(1)°·and *Z* = 2. The structure of OCP is illustrated in Fig. 2. The positions of all atoms in the region *x* = 0 to ≈ 1/4 in OCP corresponds very closely to that of the OHAp. This portion consists of two Ca^2+^ and two PO_4_^3−^ groups, corresponding to each triangular set and two Ca positions in one column in apatite, thus accounting for the Ca_6_ (PO_4_)_4_ unit. The center of inversion at 0,1/2,0 extends this region to *x* ≈ −1/4 and this region of OCP, between *x* ≈ 1/4 and *x* ≈ −1/4 has been referred to as the “apatitic layer”. Between *x* ≈ 1/4 to ≈ 3/4, the composition and the atomic positions of OCP are quite different from those of OHAp. This portion of the structure, containing the ten water molecules in the unit cell, is referred to as the “hydrated layer”. The structure of OCP has been described in terms of alternating apatitic and hydrated layers parallel to the (100) face. Closer examination of the structure reveals that the “hydrated layer” is only a hydrated region or channel along the *c*-axis at the center of the unit cell at 1/2, 1/2, 0, since the apatitic layers are held together by strong bonds involving the Ca3, Ca4, and the HPO_4_^2−^ (P5) groups along the *a*-axis.

The water molecules, O5, located near the center of the hydrated region, are not coordinated to any Ca^2+^ ion. The large thermal parameters of O5 might indicate partial occupancy at this site or disorder at several positions along this channel. The uncertainty in the numbers of water molecules in OCP may be due to the loosely bound O5 water being able to enter or leave the OCP lattice, depending on the external conditions. Regardless of the amount of O5 water, this region provides an open channel along the c-axis for the transport of Ca^2+^ or other ions that can be incorporated into the resulting apatitic products during the transformation or hydrolysis of OCP.

**Fig. 3:**
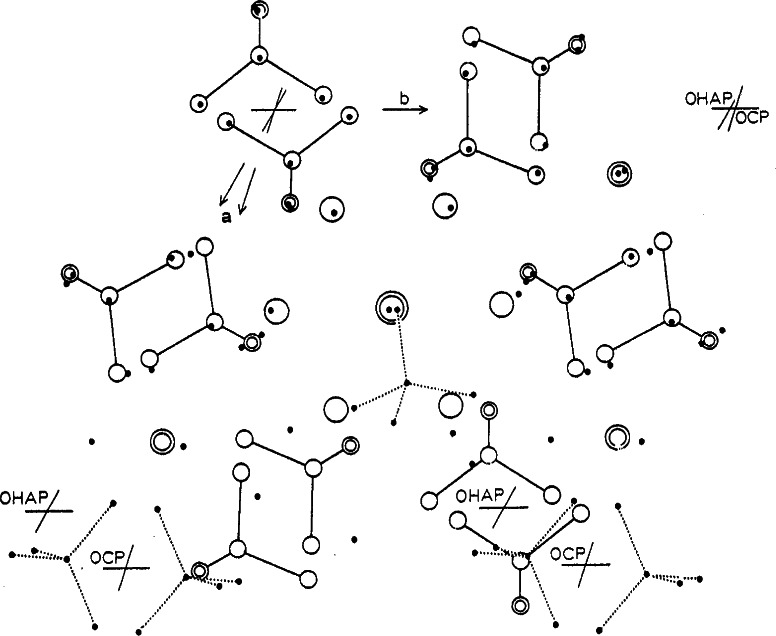
The superposition of one OHAp cell (the alternate choice indicated in Fig. 1) with half of an OCP cell projected down its *c*-axis. The open circles represent atomic positions in OHAp, while the small filled circles represent those in OCP.

**Fig. 4:**
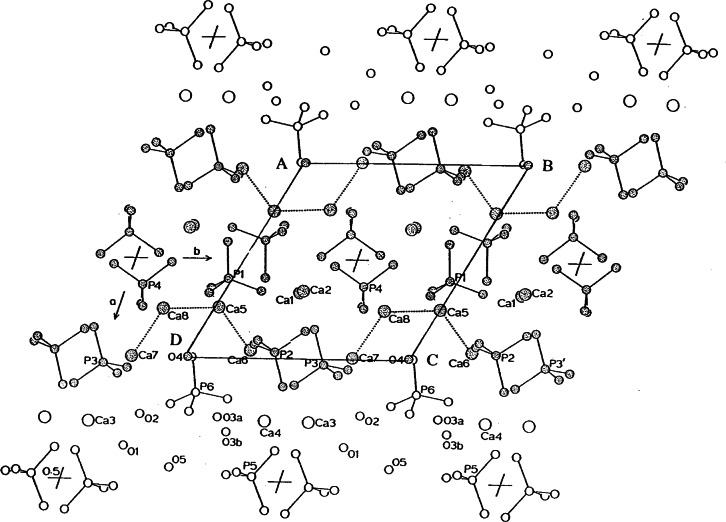
Another view of the unit cell of OCP showing the apatitic layer as the central region. A unit cell corresponding to that of hexagonal OHAp is shown. The hydrated layers on the top and bottom represent the transition state.

The overall structural relationship of the OCP and OHAp is shown in Fig. 3, where the atomic positions of OCP are superimposed on the structure of OHAp. Some similarity can be observed even in the hydrated layer. The positions of the two Ca^2+^ ions in the hydrated layer are also close to those of the column Ca^2+^ ions in apatite. Another notable feature is the location of the water molecule (O4) and one oxygen atom of the HPO_4_^2−^ at the junction of the apatitic and hydrated layers (*x* ≈ 1/4). They correspond to pivotal positions of the OH^−·^ ions at the corners of the OHAp unit cell (at *z* ≈ 1/4 and ≈ 3/4). The importance of this feature and the closeness of the fit in the structures of OCP and OHAp can be seen if the complete “apatitic layer” of OCP is compared with a unit cell of OHAp, as shown in Fig. 4. If O4 occupies a pair of corners, A and B at *z* = 1/4, the oxygen of the HPO_4_^2−^ will be at *z* = 3/4 at these corners. The positions will be reverse for the corners at C and D. For crystals to grow as OHAp, all these corner positions (ABCD at both *z* = 1/4 and 3/4) must be occupied by OH^−·^ ions instead of water and HPO_4_^2−^. While the water molecule can be easily visualized at these sites in OHAp there is no room for the HPO_4_^2−^. The pseudo-apatitic unit (ABCD, Fig. 4) containing the water molecules incorporated into true OHAp crystals offers a plausible route accounting for the lattice water molecules found in apatite. Bond valence calculations have indicated that the water molecule, O4 in OCP, is likely to be at least partially substituted by an OH^−·^ ion [14]. This view is also supported by NMR studies showing the presence of OH^−^ ions in the apatitic layer of OCP [15]. Existence of polymorphs of OCP attributed to the possible differences in bonding between the HPO_4_^2−^ ions and water molecules [16] may also be related to the possible disorder/substitution of O4 and/or O5 water molecules.

The closeness of the fit in the structures of OCP and OHAp has been used to account for the epitaxial growth and the formation of interlayered or lamellar mixtures by these two compounds [17]. Investigations of the structural models have predicted epitaxial growth of one compound on the other [18]. X-ray diffraction study of a calcium phosphate crystal that had optical properties intermediate between those of OCP and OHAp was found to diffract as independent crystals of OCP and OHAp with their *b*- and *c*-axes collinear [17]. However, when OCP and OHAp layers are very thin and random, they are characterized as interlayered mixtures of OCP and OHAp. The diffraction peaks interact with one another so that the positions of the (*h*00) peaks shift with the Ca/P molar ratio of the interlayered crystals [19]. The basic structure of the lamellar mixed crystals consists of apatitic lamellas sandwiching an OCP lamella [20].

The existence of interlayered mixtures of OCP and OHAp has been used to provide insights into the biomineralization processes, such as properties and nonstoichiometry of biological apatites, and a possible structural model for amorphous calcium phosphate [21,22]. The transition layer of OHAp to the aqueous phase has been considered to be equivalent to approximately half a unit cell of OCP on the surface of the {100} faces of OHAp. However, the possible presence of a complete pseudo-apatitic unit, ABCD (Fig. 4) in OCP itself offers another interesting possibility. While the {100} faces AB and CD would appear to present a different hydration of the OHAp surface, they are still possible candidates for epitaxial growth of OCP, as described above. From the structural point of view, the faces BC and AD are identical to the faces AB and CD and therefore, are also possible candidates for epitaxial growth of OCP. If all the faces of the pseudo-apatitic unit ABCD are hydrated, this completely hydrated unit may be used as a possible model for ACP and/or for hydrated tricalcium phosphates, which also exhibit an apatitic x-ray diffraction pattern.

### 2.3 Tetracalcium Phosphate

Tetracalcium phosphate (TTCP), Ca_4_(PO_4_)_2_O, is monoclinic, space group *P*2_1_, with unit cell parameters *a* = 7.023(1) Å, *b* = 11.986(4) Å, *c* = 9.473(2) Å and *β*= 90.90(1)°·[23]. The Ca^2+^ and PO_4_^3−^ ions in TTCP are located in four sheets perpendicular to the *b*-axis. Each sheet contains two Ca-PO_4_ columns and one Ca-Ca column. The arrangement of these columns is similar to those in glaserite where the oxide ions are extra. However, two adjacent sheets in TTCP form a layer that is closely related to that of apatite. TTCP is easily hydrolyzed in presence of DCPD or DCPA and water to form OHAp as the major ingredient of self-setting calcium phosphate cement developed in our laboratories [24] and used for repairing bone defects and cranial defects.

### 2.4 Tricalcium Phosphates

There are four polymorphs of anhydrous TCP: *α*-Ca_3_(PO_4_)_2_ (*α* -TCP), the stable phase between 1120 °C and 1470 °C, but metastable below 1120 °C; *α'*-TCP, stable above 1470 °C; *β*-Ca_3_(PO_4_)_2_ (*β*-TCP) stable below 1120 °C and *β'*-TCP stable at high pressures [25–27]. None of these compounds is known to form in biological systems. However, their relevance to biomineralization is clear considering the facts that *α*-TCP is easily hydrolyzed to OCP and that the mineral whitlockite, which is found in many biological mineralizations, has a structure very similar to that of *β*-TCP [28]. Although there are references in the literature [29] to hydrated tricalcium phosphates, they have not been clearly identified as discrete crystalline compounds.

#### 2.4.1 Glaserite-Type Structures

Structures of *α*- and *β*-TCP have been classified as glaserite-type, named after the mineral glaserite, K_3_Na(SO_4_)_2_ [30]. In glaserite, the cations and anions are arranged in two types of columns in a hexagonal arrangement, one containing only cations, Na^+^ and K^+^ (type I) and the other both cations and anions, K^+^ and SO_4_^2−^ (type II). Since the ions in each column are tightly bound along this direction, the structure has been considered as hexagonal packing of rods [31]. There are twice as many type II columns as type I. Each type I rod is surrounded by six type II rods, and each type II rod by alternate type I and type II rods. Glaserite has a cation-anion ratio of 2:1. Although many of the glaserite related structures do not satisfy this condition, the discrepancy can be accounted for by imputed ionic vacancies along selected columns.

CaK_3_H(PO_4_)_2_ can be considered to have the closest similarity to the glaserite structure. Since the radius ratio of the cations in CaK_3_H(PO_4_)_2_ is nearly identical to that of glaserite and the 2:1 ratio of cations and anions is maintained, the presence of the proton on the PO_4_^3−^ group causes only very minor structural changes [32].

**Fig. 5:**
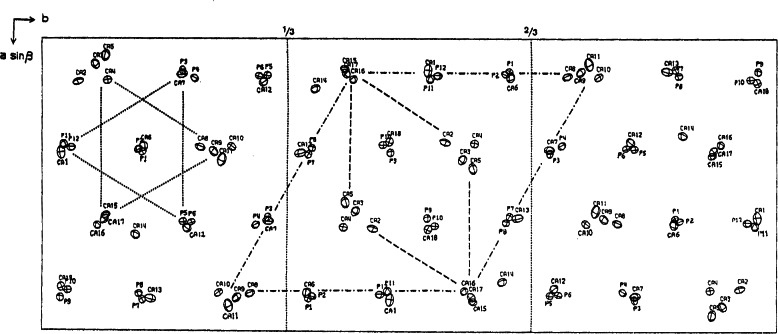
A projection of the structure of *α*-Ca_3_(PO_4_)_2_ on the (001) plane to show the columnar arrangement. Oxygen atoms of the PO_4_^3−^ groups have been omitted for clarity. The dashed lines in the center outline a cell corresponding to that of glaserite, while the dashed-dotted lines correspond to that of OHAp.

*α*-TCP crystallizes in the monoclinic space group *P*2_1_/*a* with *a* = 12.887(2) Å, *b* = 27.280(4) Å, *c* = 15.219(2) Å, *β*= 126.20(1)°, *Z* = 24 [33]. The Ca^2+^ and PO_4_^3−^ ions are packed in two kinds of columns along the *c*-axis, one containing only Ca^2+^ and the other both Ca^2+^ and PO_4_^3−^ ions in the ratio 1:2 (Fig. 5). All columns are distorted from linearity. The arrangement of these columns in a pseudohexagonal form is similar to that of glaserite. However, since *α*-TCP does not have a 2:1 cation-anion ratio, its emulation of the glaserite structure requires cation vacancies with the formula Ca_3_□(PO_4_)_2_ where □ = vacancy. All the cation vacancies are in the cation anion columns only, with the sequence ^⋯^ P–□–P–Ca^…^. The detailed environments of the Ca^2+^ ions in *α*-TCP are quite different from those of the cations in glaserite, as expected from the different sizes of the ions in the two structures and the vacancies.

*β*-TCP crystallizes in the rhombohedral space group *R*3*c* with unit cell parameters *a* = 10.439(1) Å, *c* = 37.375(6) Å, *Z* = 21 (hexagonal setting) [34]. The structure of *β*-TCP has been described as a distorted version of the Ba_3_(PO_4_)_2_ structure which has identical columns of PO_4_-Ba-Ba-Ba-PO_4_ in a hexagonal arrangement. However, since the Ca^2+^ ion is too small to provide ideal Ba_3_(PO_4_)_2_-type packing, one out of every eight formula units is missing in *β*-TCP and therefore vacancies occur at both cation and anion sites. In *β*-TCP the columns are split into two types, both containing cations and anions. Type I retains columns similar to the Ba_3_(PO_4_)_2_ structure. The other, type II, has vacancies at both anion and cation sites. Each type II column is surrounded by six type I columns; each type I column is surrounded by four type II and two type I columns. A major difference in the structures of *α*- and *β*-TCP is that there are no cation-cation columns in the *β* form.

#### 2.4.2 Mg Substitutions in TCP

Incorporation of Mg^2+^ into *α*- and *β*-TCP produces some interesting structural features. A small amount of Mg^2+^ stabilizes the *β*-TCP structure. Since the x-ray powder patterns of whitlockite and *β*-TCP or Mg-containing *β*-TCP are not easily distinguished, the names have been used interchangeably and synonymously. However, the structural studies have shown that the mineral whitlockite has a formula that approximates Ca_18_(Mg,Fe)_2_H_2_(PO_4_)_14_ and that the structure is very closely related to *β*-TCP [28]. The incorporation of Mg^2+^ into *β*-TCP and Mg^2+^ and HPO_4_^2−^ substitutions in whitlockite take place in type II columns with vacant sites. Increased substitution of Mg^2+^ also stabilizes the *α*-TCP structure, as indicated by the structural study of Ca_7_Mg_9_(CaMg)_2_(PO_4_)_12_ [35] which is closely related to *α*-TCP. The incorporation of Mg^2+^ into *α*-TCP shows that substitution occurs at the cation sites in the cation-anion columns.

#### 2.4.3 Amorphous Calcium Phosphate

Amorphous calcium phosphate (ACP) based on the generally considered molecular formula, Ca_3_(PO_4_)_2_·nH_2_O [36] may also be included as a tricalcium phosphate. Although there is no conclusive evidence for ACP as an integral mineral component in hard tissues, it plays a special role probably as a transient phase in biomineralization. In solution, ACP is readily converted to stable crystalline phases such as OCP or apatitic products. Based on radial distribution analysis of the x-ray diffraction profile it has been proposed that the basic unit of ACP consists of a 9.5 Å diameter, roughly spherical, cluster of ions Ca_9_(PO_4_)_6_ corresponding to the central apatite region [37]. The water molecules occupy the inter-cluster spaces between the aggregates of these clusters. However, Extended X-Ray Absorption Fine Structure (EXAFS) studies indicate that local order is only around 3.0 Å [38]. Infrared analysis also shows a similar lack of crystalline order [39]. This apparent lack of crystallinity is one of the more striking features of ACP. Yet the constancy in the composition of ACP over a wide range of solution conditions suggests a well-defined structural unit [40].

### 2.5 Ca-PO_4_ Sheets Containing Compounds

**Fig. 6:**
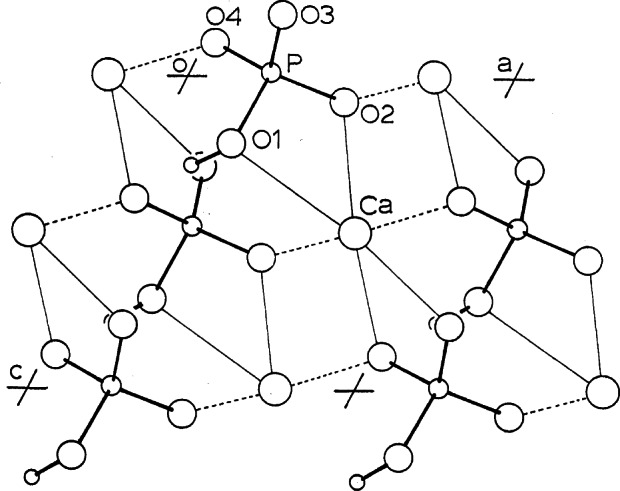
Crystal structure of DCPD as viewed down the *b*-axis. The Ca^2+^ and HPO_4_^2−^ ions are linked together to form linear chains along [101]. The linkages between chains are indicated by dashed lines. The Ca^2+^-HPO_4_^2−^ chains are stacked in a zig-zag fashion, forming corrugated sheets parallel to (010). There are two sheets per unit cell, but only one is shown. The two water molecules occupy the interstitial space between the corrugated sheets, but are omitted from this figure.

Dicalcium phosphate dihydrate (DCPD), CaHPO_4_·2H_2_O, occurs as the mineral brushite and crystallizes in the monoclinic space group *Ia* with unit cell parameters *a* = 5.812(2) Å, *b* = 15.180(3) Å, *c* = 6.239(2) Å and *β* = 116.42(3)°, *Z* = 4 [41]. The opposite edges of HPO_4_^2−^ ions are linked to Ca^2+^ ions to form linear chains that are stacked in a zig-zag fashion to form corrugated sheets parallel to the (010) face (Fig. 6). The water molecules are bonded to the Ca^2+^ ion and are located between these sheets. The packing of the Ca-HPO_4_ chains or the corrugated sheets results in several possible pseudohexagonal arrangements of the Ca^2+^ and/or PO_4_^3−^ columns, reminiscent of the glaserite structure. The Ca-HPO_4_ chains or the corrugated sheets are stacked almost exactly on top of one another along the b-axis, but with a translation along the chain such that HPO_4_^2−^ groups are above and below a Ca^2+^ ion in one sheet, and similarly Ca^2+^ ions above and below an HPO_4_^2−^ group in that sheet. Although there is no direct bonding between Ca^2+^ and HPO_4_^2−^ ions between sheets, the pseudohexagonal arrangement of Ca^2+^ and HPO_4_^2−^ ions in columns (parallel to the *b*-axis) makes DCPD a potential candidate to be included in glaserite-type structures.

Dicalcium phosphate anhydrous (DCPA), CaHPO_4_, crystallizes in the triclinic space group, $P\bar{1}$, with *a* = 6.910(1) Å, *b* = 6.627(2) Å, *c* = 6.998(2) Å, *α* = 96.34(2)°, *β* = 103.82(2)°·and *γ* = 88.33(2)°, *Z* = 4 [42]. The structures of DCPD and DCPA are closely related with similar Ca-PO_4_ chains arranged in corrugated sheets.

Monocalcium phosphate occurs as the monohydrate (MCPM) and the anhydrous salt (MCPA). Both structures show the presence of Ca-(H_2_PO_4_) chains forming corrugated sheets [43,44] as in DCPD, but with severe distortions due to the presence of H_2_PO_4_^−^ ions between these sheets.

## 3. Summary

The traditional listing of glaserite-type structure covered only the tricalcium phosphates. However, a closer examination of the structures would reveal that all calcium orthophosphates, listed here, can be included as distorted glaserite type structures, but with varying degrees of distortion. The relationship of the apatite structure to that of glaserite can be seen if the triangular Ca^2+^ and PO_4_^3−^ positions are considered to be in very distorted cation-anion columns (II) in a hexagonal arrangement (Figs. 1, 5). The centers of two of the hexagonal type II rods are occupied by the column Ca^2+^ ions, while a third set of hexagonal centers is occupied by the OH^−^ ion columns instead of cation columns, thus leading to the unusual stoichiometry. Rewriting the formula as (Ca_5_□)(PO_4_)_3_ + OH (□ = vacancy) achieves the desired 2:1 ratio of ions in glaserite and the vacancies filled by the OH^−^ ions. OCP and TTCP are structurally related to apatite. The phosphates containing Ca-PO_4_ sheets also reveal some similarity to those of glaserite. Thus, it is possible that all calcium orthophosphates can be listed under glaserite type structures. Glaserite-type structure has been described [31] as one of the most versatile arrangements in mineralogical chemistry, particularly those of phosphates, silicates and sulphates which holds true for biominerals as well.
